# Dual Energy CT (DECT) Monochromatic Imaging: Added Value of Adaptive Statistical Iterative Reconstructions (ASIR) in Portal Venography

**DOI:** 10.1371/journal.pone.0156830

**Published:** 2016-06-17

**Authors:** Liqin Zhao, Sebastian Winklhofer, Rong Jiang, Xinlian Wang, Wen He

**Affiliations:** 1 Department of Radiology, Beijing Friendship Hospital, Capital Medical University, Beijing, China; 2 Institute of Diagnostic and Interventional Radiology, University Hospital Zurich, Zurich, Switzerland; Chongqing University, CHINA

## Abstract

**Objective:**

To investigate the effect of the adaptive statistical iterative reconstructions (ASIR) on image quality in portal venography by dual energy CT (DECT) imaging.

**Materials and Methods:**

DECT scans of 45 cirrhotic patients obtained in the portal venous phase were analyzed. Monochromatic images at 70keV were reconstructed with the following 4 ASIR percentages: 0%, 30%, 50%, and 70%. The image noise (IN) (standard deviation, SD) of portal vein (PV), the contrast-to-noise-ratio (CNR), and the subjective score for the sharpness of PV boundaries, and the diagnostic acceptability (DA) were obtained. The IN, CNR, and the subjective scores were compared among the four ASIR groups.

**Results:**

The IN (in HU) of PV (10.05±3.14, 9.23±3.05, 8.44±2.95 and 7.83±2.90) decreased and CNR values of PV (8.04±3.32, 8.95±3.63, 9.80±4.12 and 10.74±4.73) increased with the increase in ASIR percentage (0%, 30%, 50%, and 70%, respectively), and were statistically different for the 4 ASIR groups (p<0.05). The subjective scores showed that the sharpness of portal vein boundaries (3.13±0.59, 2.82±0.44, 2.73±0.54 and 2.07±0.54) decreased with higher ASIR percentages (p<0.05). The subjective diagnostic acceptability was highest at 30% ASIR (p<0.05).

**Conclusions:**

30% ASIR addition in DECT portal venography could improve the 70 keV monochromatic image quality.

## Introduction

Liver cirrhosis is a common and irreversible disease that requires careful clinical management. Portal hypertension as a complication of liver cirrhosis frequently results in the development of collateral vein pathways that may cause substantial morbidity and mortality [1]. Computed tomography portal venography (CTPV) is a highly accurate diagnostic tool for anatomic delineation of portal-systemic collateral pathways prior to intervention [2–3]. It allows for displaying esophageal and gastric varices, their circulation as well as additional complications in patients with portal hypertension due to liver cirrhosis. Besides, CTPV is of importance in the preoperational evaluation of the donor and receptor of liver transplantation [4]. Unfortunately, the image quality of CTPV is commonly influenced by the cardiac function and body mass index (BMI) [5–6]. Furthermore, cirrhotic patients frequently suffer from renal dysfunction, which requires the use of reduced contrast media dosage in CT examination often resulting in a decreased image quality.

Single source dual energy CT (ssDECT) obtains spectral data by the rapid alternation of the X-ray tube potential between 80 and 140kVp during scanning. This technique has several new functions compared to the conventional CT, including the generation of monochromatic images and material decomposition image and has demonstrated its potential in clinical application and research [7–12]. The monochromatic images at different photon energies (keV) have been widely used in abdominal imaging [8–12]. ssDECT can highlight iodine contrast material by use of low keV, virtual monochromatic CT reconstructions, or by iodine (water) maps. The ability to select image sets at an optimal energy level, often relatively low photon energy, has improved the conspicuity for detecting small lesions, increased the contrast between different tissues and the enhancement of vessels, as well as better analysis of the nodular components [9–10,12]. A previous study demonstrated that the spectral CT imaging could remarkably improve the contrast enhancement of portal veins in CTPV at lower photon energies [12]. However, the image noise (IN) also increased at low energies with the use of the filtered back-projection reconstruction algorithms in first generation spectral CT scanners [11–12].

The adaptive statistical iterative reconstruction (ASIR) algorithm is a image reconstruction algorithm that reduces IN by applying iterations between the raw data and image space, improving signal-to-noise ratio, while preserving the image contrast, thus generating images of higher quality at lower radiation doses than FBP [13–14].

The scanner has since been upgraded to allow the application of ASIR in spectral CT scans, thus enabling ASIR of different percentages to be used on monochromatic images.

We therefore conducted clinical ssDECT scans to evaluate if ASIR combined with virtual monochromatic ssDECT imaging could improve the image quality of CTPV over virtual monochromatic imaging alone.

## Materials and Methods

### Patient population

An ethical approval for this study was obtained from the Ethics Committee of Beijing Friendship Hospital, Capital Medical University and a written informed consent was obtained from all patients. From April 2014 to November 2014, 51 clinically confirmed cirrhotic patients were prospectively enrolled in this study. Four cases with thrombus in the main stem or both branches of the portal vein, and two with poor image quality due to respiratory motion artifacts were excluded, resulting in 45 patients who were finally included in this study. These patients included 26 cases of cirrhosis secondary to hepatitis B, three cases of cirrhosis secondary to hepatitis C, 13 of alcoholic cirrhosis, and three of primary biliary cirrhosis. The study population consisted of 28 male and 17 female patients with ages ranging from 41–81 years (mean age, 59.21±10.8). With regard to liver function in terms of the Child-Pugh classification, eight cases were grades as A, 22 graded as B, and 15 grades as C. The mean body mass index (BMI), calculated as weight in kilograms divided by height in meters squared, was 24.80±4.14.

### Imaging technique

All patients underwent three-phase [non-enhanced (NE), arterial phase (AP), and portal vein phase (PVP)] CT scans on a Discovery CT 750 HD scanner (HDCT, GE Healthcare, Milwaukee, Wisconsin, USA). Before the CT scan 600 ml of water were administered orally to the patient as negative contrast media over a 30 minute period. The NE CT was performed using conventional CT scan mode. The AP and PVP were performed using a single tube, fast dual kVp (80 kVp and 140 kVp) switching scan technique. The scan coverage of NE and AP included the diaphragmatic dome to the lower poles of both kidneys and that of the PVP was from 2 cm above the tracheal bifurcation to the lower poles of both kidneys.

The NE (conventional) CT scan parameters included: 120 kVp, auto mAs for a noise index of 12. The AP and PVP (spectral CT) scan parameters included: helical, 550 mAs, 0.8 second tube rotation time, 40 mm detector coverage, pitch factor: 0.985:1, 35 cm display field of view (DFOV). The non-ionic contrast media Iohexol (Omnipaque 350, GE healthcare, Shanghai, China) at the dose of 500 mgI/kg body weight (1.4 ml/kg) was injected in 30 seconds with a power injector (with an injecting rate between 4.0 to 5.0 ml per second) through median cubital vein. The AP scans were triggered 20 seconds after the attenuation in the descending aorta reached 100 HU on the monitoring scan. The PVP scans began 30 seconds after the end of the AP scan.

Due to the fact that 70 keV was usually used for diagnostic interpretation in our hospital, the monochromatic PVP images at 70 keV were reconstructed at 4 ASIR levels (0%, 30%, 50%, and 70%) both at 5.0 mm and 0.625 mm slice thickness on CT console by technologist. The image of 5.0 mm slice thickness was used for the measurement and that of 0.625 mm was used for CTPV reformation.

### Data processing and image analysis

#### Objective score

**Fig 1:**
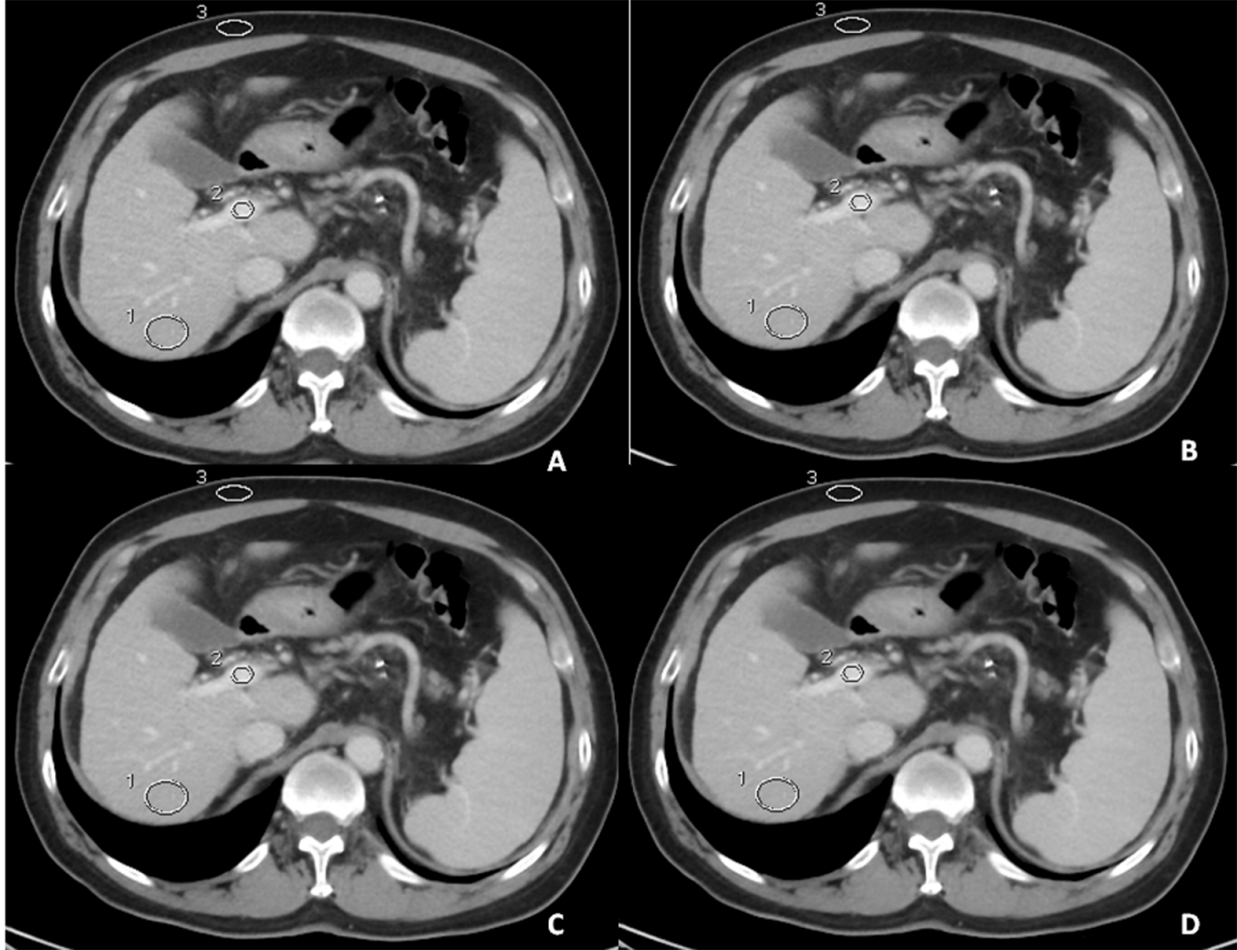
Enhanced axial images at portal venous phase of a 63-year female patient with portal hypertension secondary to liver cirrhosis with different percentage ASIRs, for objective evaluation of CT portal venography quality. **(A), (B), (C)** and (**D)** were obtained with 0%, 30%, 50%, and 70% percentage ASIR reconstruction, respectively. The marked regions were ROIs in the main stem of portal vein, liver parenchyma, and subcutaneous fat.

The four sets of images (0%, 30%,50%, and 70% ASIR) were sent to an AW4.4 workstation (GE Healthcare, Wisconsin, USA). The 5.0 mm image slice including the right branch of portal vein main stem was used for measurements. A circular or elliptic region of interest (ROI) with pixels numbers ranging from 80 to 400 was placed on different areas to measure the mean CT values and standard deviation (SD) (**Fig 1**). The SD from the ROI of the liver parenchyma was interpreted as the objective image noise (IN). These areas included the right or left intra-hepatic portal vein (mainly the right branch; the left branch was used when the right branch was occluded by a thrombus), the hepatic parenchyma of the right lobes (excluding large blood vessel) and the subcutaneous fat tissue of the abdomen. The size of ROI covered 80% of the area for the portal veins, and the mean number of pixels were 250 (range, 100–400 pixels) for the subcutaneous fat tissue and 500 (range, 450–600 pixels) for the liver parenchyma. The same ROIs setting were used for the four ASIR percentage sets by using a copy-paste function.

The contrast-to-noise-ratio (CNR) was defined according to the formula: $CNR=\frac{ROIpv-ROIl}{SDf}$, where ROIpv denotes the average CT value from the left or right intra-hepatic portal veins, ROIl denotes the average CT value of the liver parenchyma in the right lobe of the same slice, and SDf denotes the mean background IN. The SD of the subcutaneous fat tissue in the abdomen was used as the mean IN SDf. The CNR measurements were obtained for the four sets of different ASIR percentage monochromatic images.

#### Subjective score

**Fig 2:**
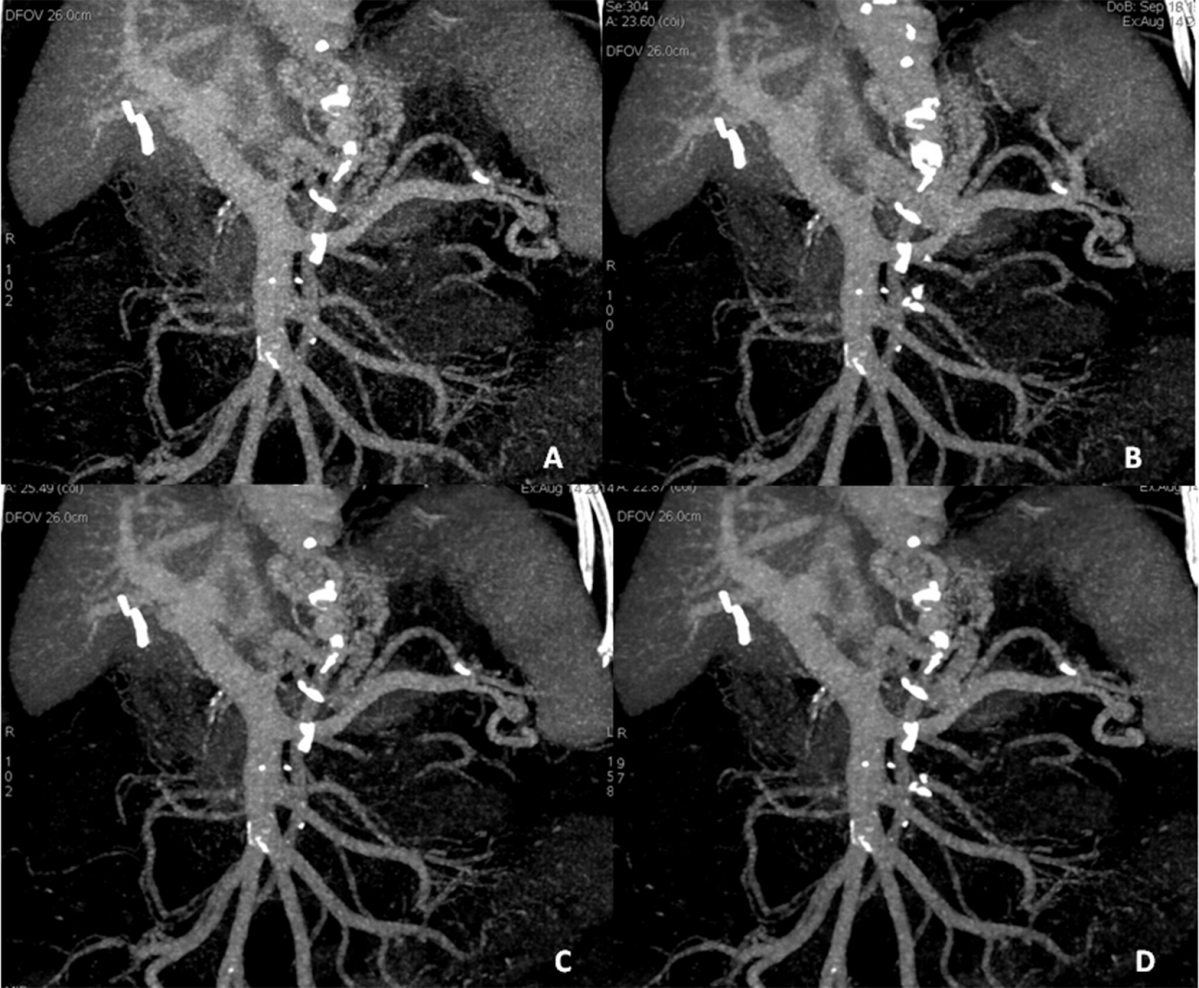
Subjective evaluation of rapid switching single source dual energy CT (ssDECT) portal venography images of a cirrhotic patient with portal hypertension secondary to liver cirrhosis with different percentage adaptive statistical iterative reconstructions (ASIRs). **(A), (B), (C)** and (**D)** were obtained with 0%, 30%, 50%, and 70% percentage ASIRs reconstruction, respectively. The same slice thickness, field of view and nearly identical angle were used for reconstruction of all images.

The four sets of axial PV images with different ASIR percentages were post-processed to form maximum intensity projections (MIP) and curved plane reformat (CPR) images (**Fig 2**). The portal vein and its intra-hepatic branches were depicted as well as the splenic veins and the superior mesenteric veins. All images were interpreted by two independent radiologists with 10 and 12 years experience in abdominal imaging. Both radiologists were blinded to the ASIR percentages of the images. Each patient’s image sets were randomly allocated to the two observers. Scores were given to these images regarding their quality of the axial and the reformatted CTPV images from the four data sets of ASIR reconstructions using the subjective criteria. Therefore a 5-point Likert scale was used to evaluate the image quality of CTPV [8] (**Table 1**). Each case was evaluated regarding the sharpness of portal vein boundaries, and diagnostic acceptability (DA) at the same window width, window level and display field of view.

**Table 1:** Grading scale for qualitative analysis of portal vein.

Grading scale	Sharpness	Diagnostic acceptability
1	Blurred	Unacceptable
2	Poor	Suboptimal
3	Average	Diagnostic
4	Better	Superior
5	Sharpest	Excellent

Note: Sharpness: the sharpness of portal vein boundaries.

### Statistical analysis

Statistical analysis was carried out using the SPSS version 17.0 software (SPSS for Windows; SPSS Chicago, IL). For quantitative data, the results are expressed as the mean ± SD. Repeated measures analysis of variance was performed on IN and CNR and the Friedman test was used to compare the scores of the sharpness of intra-hepatic PV boundaries and DA among the four different ASIR percentages groups of the 70 keV monochromatic imaging set. p<0.05 is defined as statistical significance. The Bonferroni correction was used for all multiple comparisons, P<0.008 (0.05/6, 6 groups were compared) is defined as statistical significance.

Inter-observer agreements were calculated using Cohen’s Kappa statistics.

## Results

### Objective score

Among the 45 patients, 43 measurements of PV were performed on right intra-hepatic portal vein and two were performed on left branch due to a thrombus of the right branch.

**Fig 3:**
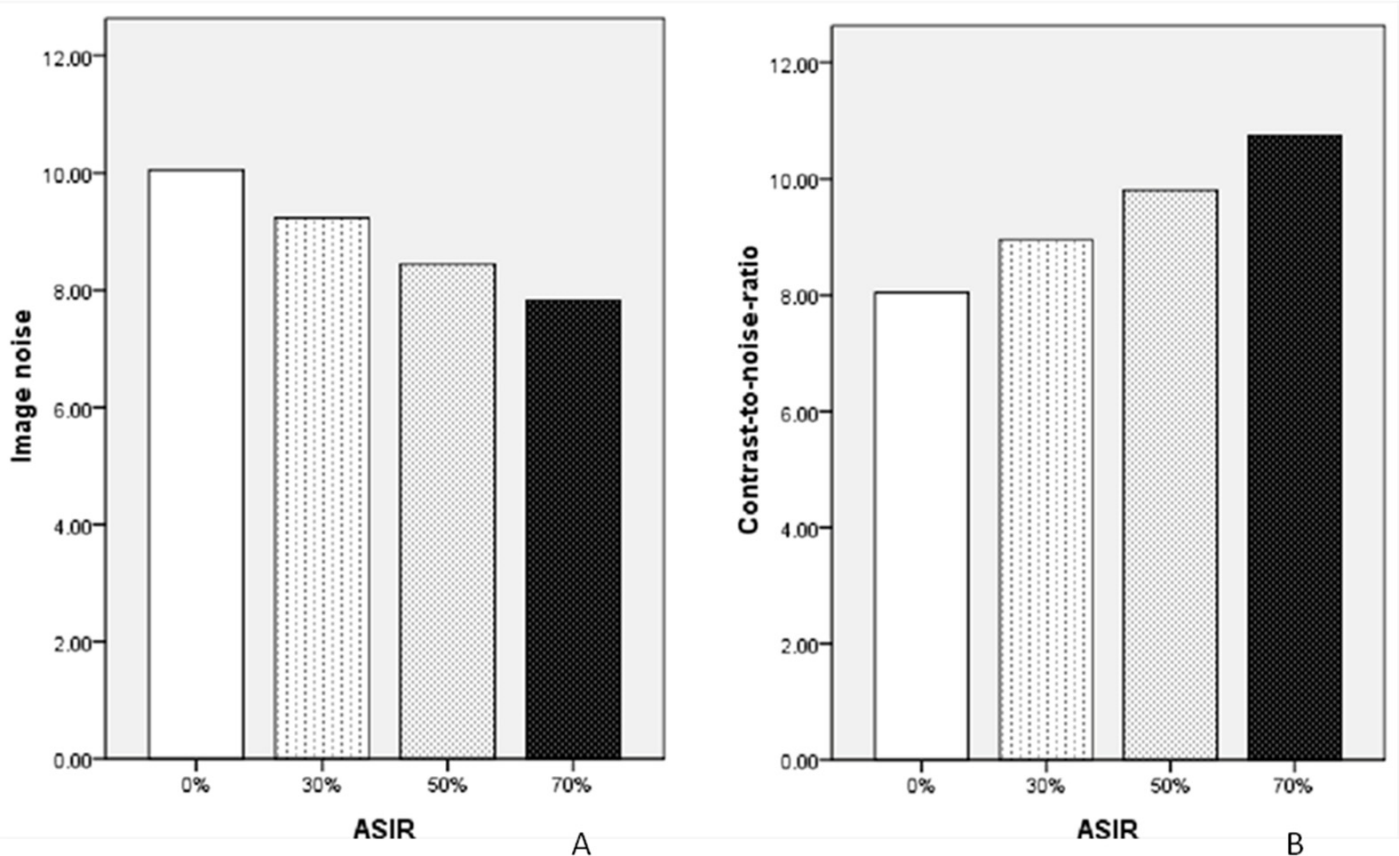
Objective image quality assessment of 70 keV monochromatic images with 0%, 30%, 50%, and 70% percentage ASIRs levels. (**A)** Comparisons of image noise (IN with the unit of HU) of portal vein. (**B)** Comparisons of the contrast-to-noise-ratio (CNR) of portal vein to liver parenchyma.

Comparisons of IN of PV, and PV-to-liver CNR among the 70 keV monochromatic images with four different ASIR percentages are shown in **Tables 2** and **3,** and **Fig 3**. The results demonstrate that the IN decreased whereas CNR increased with the increase of ASIR percentage, both with statistically significant differences (p<0.05).

**Table 2:** Objective and subjective image quality assessment with different ASIR percentages.

Groups	0% ASIR	30% ASIR	50% ASIR	70% ASIR	F value/ Chi-Square	P value
IN	10.05±3.14	9.23±3.05	8.44±2.95	7.83±2.90	222.76	<0.001*
CNR	8.04±3.32	8.95±3.63	9.80±4.12	10.74±4.73	89.39	<0.001*
Sharpness	3.13±0.59	2.82±0.44	2.73±0.50	2.07±0.54	54.97	<0.001*
DA	2.58±0.62	3.69±0.73	3.24±0.74	2.58±0.72	53.18	<0.001*

Note: ASIR: adaptive statistical iterative reconstruction; IN: image noise

CNR: contrast-to-noise-ratio; DA: diagnostic ability

Sharpness: the sharpness of portal vein boundaries

F value / Chi-Square: F value for IN and CNR; Chi-Square value for the Sharpness of portal vein boundaries and DA

* *p* value< 0.05.

**Table 3:** Comparison of image quality assessment of different ASIR percentage groups.

	0% ASIR vs 30% ASIR	0% ASIR vs 50% ASIR	0% ASIR vs 70% ASIR	30% ASIR vs 50% ASIR	30% ASIR vs 70% ASIR	50% ASIR vs 70% ASIR
IN	<0.001*	<0.001*	<0.001*	<0.001*	<0.001*	<0.001*
CNR	<0.001*	<0.001*	<0.001*	<0.001*	<0.001*	<0.001*
Sharpness	0.705	<0.001*	<0.001*	<0.001*	<0.001*	0.020
DA	<0.001*	<0.001*	0.023	0.001*	<0.001*	<0.001*

Note: ASIR: adaptive statistical iterative reconstruction; IN: image noise

CNR: contrast-to-noise-ratio; DA: diagnostic ability

Sharpness: the sharpness of portal vein boundaries

* p value< 0.008(0.05/6).

### Subjective score

**Fig 4:**
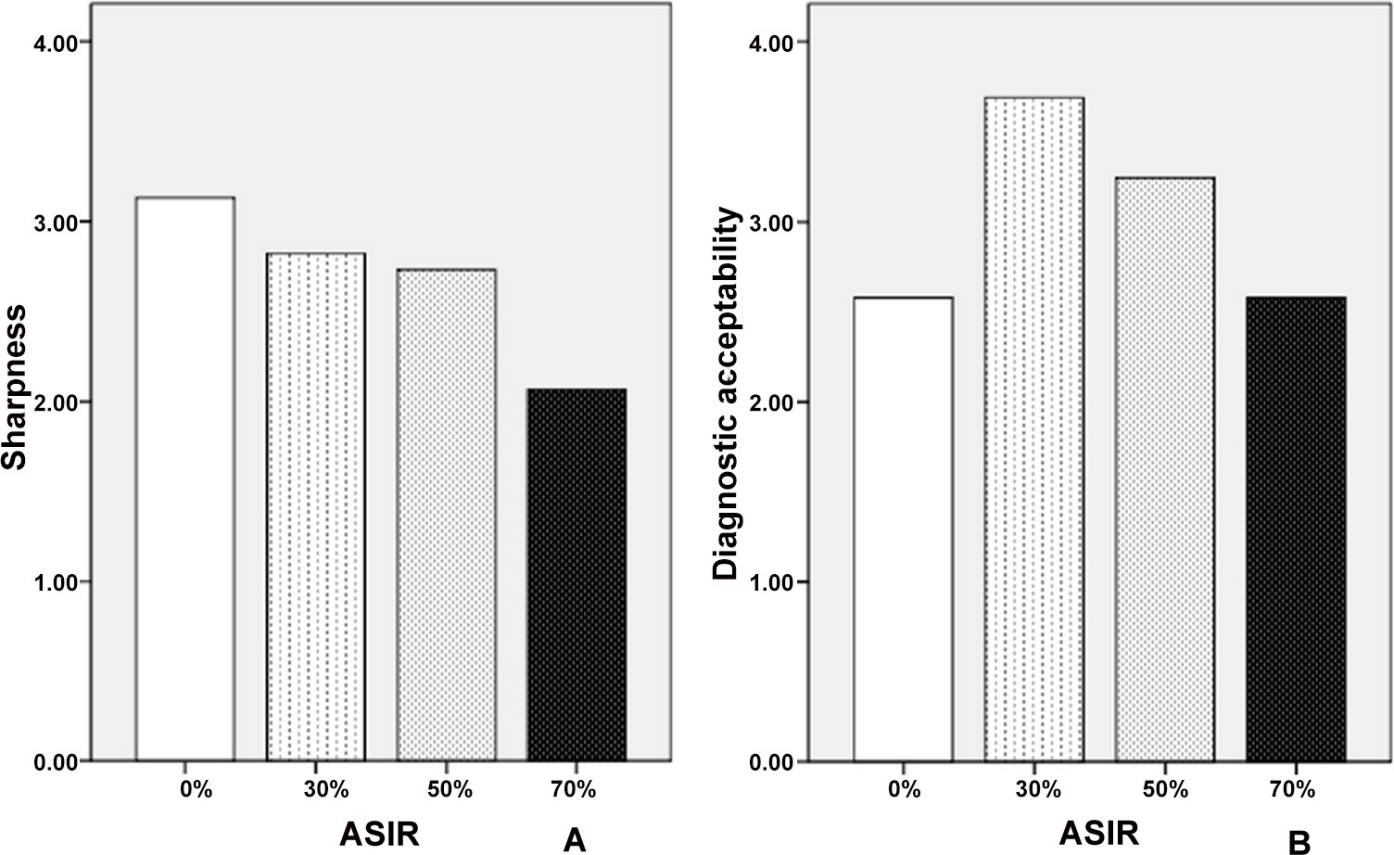
Subjective image quality assessment of the 70 keV monochromatic images with 0%, 30%, 50%, and 70% percentage ASIRs levels. (**A)** Comparisons of the sharpness of portal vein boundaries. (**B)** Comparisons of the diagnostic ability (DA).

The subjective monochromatic image scores of the four ASIR levels are shown in **Tables 2** and **3** and **Fig 4**. The sharpness of PV boundaries, and DA of four ASIR levels were all significantly different (p<0.001).

For the sharpness of PV boundaries, 0% ASIR had the highest score with the tendency that the sharpness decreased as the ASIR percentage increased, yet, the differences between 0% and 30%, 50% and 70% were not statistically significant (p value: 0.705, and 0.020, respectively). For the DA of CTPV, 30% ASIR provided the highest score (3.86, p<0.001). Apart from 0% and 70% ASIR groups, the differences between other groups were all statistically significant (p<0.001).

The kappa value for sharpness of PV boundaries, and DA of each subgroup were 0.568, 0.685 and 0.590, respectively.

## Discussion

Our study demonstrates the benefit of combining monochromatic reconstructions from ssDECT with iterative reconstructions for an improved image quality in portal venography.

Image reconstructions from ssDECT allow for obtaining data sets of 101 different monochromatic energy levels from 40 to 140keV. Lower keV images can enhance the image contrast, whereas higher keV images can decrease the beam hardening and metal artifacts [11,14]. Previous studies have demonstrated that compared to single energy CT, monochromatic image of ssDECT could significantly improve the image quality of CT angiography and visualization of vascular lesions and vessels with contrast enhancement, such as the pulmonary artery, coronary artery, lower extremity artery, hepatic vein, and portal vein [12, 15–18].

A previous study on the CTPV has shown that monochromatic image at 51keV could increase the CNR by about 100% when compared to polychromatic images, but also increased the IN by about 30% [12]. Clinically, liver cirrhosis is frequently accompanied by ascites, resulting in an inevitably increase of IN and further decrease of image quality. Therefore, if the IN could be reduced while improving the image CNR, the clinical use of DECT could be broaden.

ASIR is one of the recently introduced iterative reconstruction algorithms in CT. It uses a statistical model to reduce the IN during the reconstruction process [15, 19], and has the option to provide different ASIR strengths by blending with the conventional filtered back-projection (FBP) reconstruction with different ASIR percentages [19]. For example, 30% ASIR is 30% ASIR reconstruction mixed with 70% FBP. The ASIR percentage can be selected according to the examination purpose. In our study, 0%, 30%, 50% and 70% ASIR were selected for the monochromatic image (0% is equivalent to FBP reconstruction algorithm). We stopped at 70% percentage based on a previous study which showed that the CNR and the subjective evaluation on image quality with ASIR percentages more than 70% was worse [20–21], whereas 40–50% was preferred for the image quality [21].

The previous generation of DECT used FBP as the standard reconstruction, which failed to take the system noise model into account, so its noise level was high, especially for images at low photon energies where photon numbers were scarce [22]. The integration of ASIR with the dual-energy spectral CT imaging model provides the capability of further reducing IN in DECT. Our results showed, that compared to FBP reconstruction (0% ASIR), ASIR could reduce IN and thus improve image quality. The degree of noise reduction increased with the increase of ASIR percentage. For example, compared to FBP, the 30% ASIR reconstruction decreased IN by 10.2%, whereas 20.5% and 29.8% IN reduction was achieved with 50% ASIR and 70% ASIR, respectively.

However, it does not necessary mean that higher ASIR percentage always produces better image quality. Our subjective evaluation results showed that monochromatic images with 30% ASIR had the best diagnostic acceptability, which is similar to the previous phantom and clinical studies results [21].

The possible reason for the inferior subjective score of 70% ASIR to that of 50% ASIR may be due to the fact that higher percentage ASIR could over-smooth the margin of the image structure and reduce the contrast between different tissues and thus decrease the image acceptability. In our study the texture deformation was not observed for CTPV in the subjective evaluation, but it did exist in some of the liver low contrast lesions when high percentage (70%) ASIR was applied.

The improvement of image quality in spectral CT angiography comes from the fact that many various sets of monochromatic images can be obtained while post-processing. Among them, there will be a certain keV which could display the best CNR between two tissues. The optimal keV levels are different according to the targets [9–12]. Previous studies have shown that the monochromatic image at 51 keV level was optimal for displaying portal vein [12]; whereas 70 or 78 keV images are often used for diagnostic interpretation [11]. For our study we have chosen a 70 keV setting.

This study has several limitations. First, we could not make a comparative analysis between spectral CT and conventional CT at different ASIR percentages. Though previous studies have verified that the image quality of gemstone spectral imaging (GSI) was superior to that of the conventional CT [17], they were performed without ASIR reconstruction, and our study was focused on the addition of ASIR in monochromatic spectral CT scans. Second, we did not take into account the potential effect of the BMI of patients even though the effect of BMI should be minor theoretically. A previous study on the abdominal image quality by using low dose ASIR reconstruction and normal dose FBP reconstruction has shown that the sharpness of structure boundaries of low dose ASIR reconstruction in patients with low BMI was decreased, although diagnostic acceptability was nearly identical to those for routine-dose CT with FBP [23]. Third, we didn’t use the power noise spectrum on the objective evaluation of interatively reconstructed CT images. The noise power spectrum for the iterative reconstruction algorithm is a very important parameter and should be investigated when the algorithm is developed. However, it is less important in our study since the aim of our study was more focused on the resolving of clinical problems, and relative performance between the different strengths of the iterative reconstruction was investigated.

In conclusion, our study has shown that monochromatic spectral CT images reconstructed with ASIR can reduce IN and improve image quality. With a proper ASIR percentage (30%), one could maintain the superior contrast enhancement advantage of ssDECT imaging for hepatic diseases, and balance image CNR, spatial resolution and DA.

## Supporting Information

S1 File Raw data of objective and subjective image quality assessment of portal vein with different ASIR percentages. (PDF)
