# Supplementary material for: Dual Energy CT (DECT) Monochromatic Imaging: Added Value of Adaptive Statistical Iterative Reconstructions (ASIR) in Portal Venography
Source: PLoS One. 2016 Jun 17;11(6):e0156830. doi: 10.1371/journal.pone.0156830 (PMC4912087; doi:10.1371/journal.pone.0156830)
Supplement: S1 File — (PDF) [file pone.0156830.s001.pdf]

name	gender	BMI	CT_LIVER_700	CT_LIVER_7030	CT_LIVER_7050	CT_LIVER_7070
1	1	20.43	127.63	127.61	127.69	127.77
2	2	29.14	114.79	114.75	114.77	114.8
3	2	24.45	117.99	117.98	117.96	117.99
4	1	25.83	106.75	106.61	106.09	107.05
5	1	20	123.96	123.95	123.95	123.94
6	1	25.26	128.09	128.13	128.17	128.11
7	1	28.37	108.7	108.6	108.6	108.57
8	2	27.18	122.67	122.56	122.5	122.44
9	1	26.03	117.77	117.8	117.82	117.81
10	2	21.94	96.08	96.14	96.18	96.24
11	2	24.34	129.51	129.53	129.57	129.64
12	1	24.22	122.86	122.81	122.84	122.84
13	1	26.17	99.47	99.47	99.41	99.45
14	1	21.36	100.32	100.29	100.2	100.16
15	1	31.38	117.44	117.48	117.41	117.43
16	1	29.41	158.8	158.75	158.6	158.53
17	2	19.59	127.02	127	127.02	126.97
18	1	32.47	151.14	151.16	151.24	151.22
19	2	22.49	92.97	92.97	92.94	92.94
20	2	26.4	124.6	124.66	124.71	124.71
21	2	26.57	132.57	132.56	132.56	132.62
22	1	26.84	109.57	109.51	109.54	109.56
23	1	23.88	117.99	117.99	117.98	117.99
24	1	21.48	97.86	97.82	97.81	97.86
25	1	19.61	138.38	138.29	138.2	138.24
26	2	26.12	98.12	98.03	98.06	98.05
27	2	34.6	146.77	146.66	146.49	146.38
28	1	17.72	139.22	139.14	139.09	139.02
29	2	26.9	137.35	137.24	137.26	137.25
30	1	20.7	116.41	116.45	116.34	116.3
31	2	22.31	107.9	107.9	107.9	107.9
32	1	31.25	143.16	143.22	143.24	143.28
33	1	20.96	97.91	97.87	97.85	97.83
34	2	19.53	119.79	119.83	119.84	119.87
35	2	20.52	122.54	122.52	122.58	122.61
36	1	23.53	107.5	107.34	107.27	107.2
37	1	23.67	134.55	134.44	134.48	134.5
38	2	32.11	102.53	102.5	102.4	102.39
39	1	25.1	155.04	155.08	155.05	155.15
40	2	22.86	95.98	95.95	96	96
41	1	21.78	108	107.98	108	108
42	1	25.61	113.4	113.38	113.46	113.6
43	1	25.39	119.5	119.5	119.5	119.52
44	1	23.31	108	108	108	108.2
45	1	31.51	102.6	102	102	102.47

HU_PV_700	HU_PV_7030	HU_PV_7050	HU_PV_7070	SD_FAT_700	SD_FAT_7030	SD_FAT_7050
240.41	240.12	239.6	239.29	6.15	5.4	4.78
196.56	196.05	195.52	195.38	7.63	7.02	6.37
168.06	167.71	167.22	166.74	6.11	5.36	4.65
158.46	158.17	157.67	157.33	7.89	7.3	6.61
204.92	204.3	203.72	203.89	6.01	5.27	4.49
186.18	185.74	185.38	185.01	7.15	6.41	5.72
154.43	153.94	153.54	153.23	9.12	8.11	7.1
204.8	203.67	203.22	202.9	11.04	10	9
221.79	221.17	220.75	221.12	7.17	6.42	5.76
138.93	138.65	138.44	138.18	8.13	7.61	7.16
190.55	190.25	190.08	189.7	8.04	7.4	6.87
194.05	193.57	193.14	192.57	6.75	5.9	5.18
167.29	167.16	166.86	166.97	7.14	6.66	5.61
168.96	168.6	168.31	168.01	7.34	6.95	6.55
177.58	177.34	177.11	176.88	13.65	13.1	12.47
207.87	207.7	207.64	206.97	7.39	6.74	6.07
181.12	180.96	180.8	180.6	10.97	6.05	5.37
232.25	231.96	231.82	231.58	10.14	9.7	9.29
147.88	147.63	147.38	147.1	6.87	6.07	5.29
205.09	204.66	204.14	203.63	7.67	6.95	6.28
243.94	242.06	241.62	240.72	6.84	6.38	6
191.61	191.52	191.23	190.68	9.27	8.51	7.96
155.39	155.11	154.9	154.7	6.63	5.98	5.31
192.18	191.84	191.48	190.95	14.23	7.04	6.45
155.13	154.93	154.82	154.65	9.08	8.49	7.89
160.24	159.94	159.75	159.45	9.22	8.43	7.69
181.49	181.03	180.64	180.17	9.25	8.24	7.33
192.82	192.9	192.96	192.52	8.16	7.49	6.84
241.32	239.89	238	235.86	8.21	7.6	7.04
178.81	178.32	177.9	177.42	8.34	7.88	7.41
142.76	142.11	141.65	140.93	10.76	10	9.37
150.71	150.25	149.55	148.42	11.19	10.48	9.92
232.49	231.84	230.93	230.12	8.48	7.57	7.15
133.75	133.44	133.19	133.19	6.03	5.39	4.74
163.09	162.69	162.18	161.69	7.22	6.78	6.2
216.06	215.85	215.68	215.4	8.29	7.96	7.5
193.33	192.93	192.81	192.64	10.36	9.63	8.93
177.14	176.91	176.47	176.11	16.17	15.27	14.54
187.11	186.55	186.05	185	8.1	7.75	7.25
155.2	155.58	155.34	155.3	7.85	7.25	6.63
146.17	146.87	147.28	147	10.31	9.64	8.85
184.2	193.9	183.6	182.98	11.36	10.61	9.98
240	239.6	238.8	238.4	8.54	7.4	6.69
207.55	206.9	206.35	205.8	8.91	8.31	7.8
162.7	161.98	161.76	161.48	8.45	7.43	6.36

SD_FAT_7070	CNR_7030	CNR_7050	CNR_7070	IN_LIVER_700	IN_LIVER_7030	IN_LIVER_7050
4.16	20.84	23.41	26.81	7.73	7.36	6.71
5.79	11.58	12.68	13.92	9.59	8.71	7.9
3.97	9.28	10.59	12.28	8.04	6.97	5.98
5.96	7.06	7.8	8.44	11.19	10.11	9.11
4.01	15.25	17.77	19.94	4.53	3.87	3.25
5.07	8.99	10	11.22	10.94	9.74	8.64
6.25	5.59	6.33	7.15	9.26	8.3	7.34
8.05	8.11	8.97	10	10.41	9.25	8.16
5.12	16.1	17.87	20.18	11.61	10.68	9.76
6.78	5.59	5.9	6.19	8.82	7.77	6.75
6.41	8.21	8.81	9.37	10.01	8.95	7.92
4.43	11.99	13.57	15.74	5.71	5.11	4.58
5.26	10.16	12.02	12.84	7.67	6.75	5.13
6.36	9.83	10.4	10.67	7.4	6.53	5.71
11.85	6.46	6.72	7	13.58	12.23	10.95
5.46	7.26	8.08	8.87	9.29	8.37	7.41
4.75	8.92	10.01	11.29	9.49	8.45	7.44
8.82	8.33	8.67	9.11	8.59	7.81	7.04
4.61	9	10.29	11.75	7.1	6.3	5.52
5.7	10.92	11.99	13.15	11.78	10.76	9.81
5.63	11.3	11.93	12.61	7.6	6.83	6.13
7.26	9.64	10.26	11.17	9.23	8.49	7.82
4.75	6.18	6.94	7.72	6.96	6.13	5.31
5.86	8.82	9.6	10.51	8.26	7.23	6.21
7.36	5.03	5.38	5.7	5.03	5.03	4.46
6.92	11.25	12.34	13.65	15.26	13.93	12.63
6.32	11.31	12.48	14.16	14.53	13.03	11.56
6.18	5.23	5.67	6.21	5.38	4.72	4.14
6.53	12.45	13.31	14.22	8.58	7.77	6.83
7.04	2.16	2.27	2.4	7.7	7.08	6.51
8.74	5.48	5.79	6.15	6.81	6.17	5.5
9.46	6.93	7.3	7.62	11.02	9.92	8.98
6.73	12.56	13.28	14.09	7.44	6.53	5.69
4.2	10.59	11.95	13.39	5.57	4.98	4.41
5.73	9.44	10.24	10.89	6.25	5.68	5.09
7.05	6.06	6.41	6.82	7.25	6.35	5.71
8.26	1.29	1.43	1.51	13	11.78	10.5
13.7	5.99	5.58	5.88	11.1	10	9.1
6.85	10.91	11.55	12.15	9.69	8.82	7.96
6.05	15.3	16.64	18.15	7.36	6.64	5.95
8.13	5.6	6.07	6.58	8.7	7.89	7.05
9.64	6.34	6.69	6.88	6.89	6.34	5.8
5.94	6.3	6.92	7.66	6.87	6.18	5.55
7.26	8.01	8.49	9.05	8.45	7.58	6.76
5.34	9.21	10.67	12.54	5.46	4.74	4.04

_LIVER_70	IN_PV_700	IN_PV_7030	IN_PV_7050	IN_PV_7070	HU_PV_700	HU_PV_7030	HU_PV_7050
6.14	6.61	6.06	5.53	5.11	240.41	240.12	239.6
7.1	10.78	9.78	8.6	7.6	196.56	196.05	195.52
5.01	9.14	8.18	7.34	6.62	168.06	167.71	167.22
8.11	13.44	12.25	11.03	9.85	158.46	158.17	157.67
3.01	8.3	8.03	7.68	7.22	204.92	204.3	203.72
7.53	9.93	8.8	7.76	6.72	186.18	185.74	185.38
6.44	12.08	11.13	9.07	8.03	154.43	153.94	153.54
7.02	13.47	12.13	11.45	10.72	204.8	203.67	203.22
8.86	11.03	10.13	9.42	8.82	221.79	221.17	220.75
5.79	9.2	8.09	7.2	6.26	138.93	138.65	138.44
7	9.75	8.86	8.1	7.32	190.55	190.25	190.08
4.01	8.04	7.45	7.03	6.77	194.05	193.57	193.14
4.65	6.75	5.8	5.04	4.32	167.29	167.16	166.86
4.96	7.19	6.39	5.56	4.88	168.96	168.6	168.31
9.66	12.15	10.92	9.71	8.94	177.58	177.34	177.11
6.55	9.65	8.94	8.19	7.7	207.87	207.7	207.64
6.48	8.83	7.71	6.85	8.83	181.12	180.96	180.8
6.34	10.73	10.27	9.54	9.01	232.25	231.96	231.82
4.83	5.58	5.02	4.45	4.03	147.88	147.63	147.38
8.89	10.65	9.63	8.63	7.73	205.09	204.66	204.14
5.45	10.04	9.47	8.93	8.46	243.94	242.06	241.62
7.16	11.53	10.87	10.54	10.38	191.61	191.52	191.23
4.56	8.23	7.27	6.37	5.47	155.39	155.11	154.9
5.24	6.9	6.03	5.28	4.54	192.18	191.84	191.48
3.9	6.14	5.37	4.7	4.06	155.13	154.93	154.82
11.34	15.64	14.43	12.9	11.63	160.24	159.94	159.75
10.19	17.83	16.31	15.21	13.78	181.49	181.03	180.64
3.58	6.57	6	5.56	5.16	192.82	192.9	192.96
6.01	20.12	19.96	19.4	19.14	241.32	239.89	238
5.98	11.05	10.58	10.11	9.68	178.81	178.32	177.9
5.03	8.76	8.35	7.92	7.51	142.76	142.11	141.65
8.04	12.71	11.69	10.76	9.91	150.71	150.25	149.55
4.93	8.35	7.55	6.69	5.92	232.49	231.84	230.93
3.91	7	6.53	6	5.81	133.75	133.44	133.19
4.53	6.77	6.44	6	5.73	163.09	162.69	162.18
5.08	11.1	10.09	9.79	9.49	216.06	215.85	215.68
9.2	11.23	10.16	9.27	8.5	193.33	192.93	192.81
8.12	14.97	13.68	12.35	11.01	177.14	176.91	176.47
7.18	13	12.6	11.6	11.18	187.11	186.55	186.05
5.3	7.68	7.2	6.64	6.13	155.2	155.58	155.34
6.28	10.31	9.34	8.45	7.58	146.17	146.87	147.28
5.28	6.45	5.74	5.18	4.52	184.2	193.9	183.6
4.94	11.02	10.51	10.16	9.9	240	239.6	238.8
5.95	8.95	8	7.08	6.19	207.55	206.9	206.35
3.28	6.48	5.69	4.88	4	162.7	161.98	161.76

HU_PV_7070	sharpness_700	sharpness_7030	sharpness_7050	sharpness_7070	DA_700
239.29	4	3	3	2	3
195.38	3	3	3	3	3
166.74	3	2	2	2	3
157.33	3	3	3	3	2
203.89	3	3	3	3	2
185.01	4	4	4	3	3
153.23	4	3	3	2	3
202.9	3	3	3	2	3
221.12	3	3	3	2	3
138.18	3	3	3	2	3
189.7	3	3	3	2	2
192.57	3	3	3	2	2
166.97	3	3	3	2	3
168.01	3	3	3	2	3
176.88	2	2	2	1	1
206.97	3	2	2	1	2
180.6	4	3	3	2	3
231.58	3	3	3	3	2
147.1	3	3	3	3	2
203.63	4	3	3	3	3
240.72	4	3	3	2	3
190.68	4	3	3	2	3
154.7	3	3	3	2	3
190.95	2	2	2	2	3
154.65	3	3	2	2	2
159.45	3	3	3	2	2
180.17	3	3	3	2	3
192.52	3	3	3	2	3
235.86	3	3	3	2	3
177.42	3	3	3	2	3
140.93	3	2	2	1	2
148.42	4	3	3	2	3
230.12	3	3	2	2	3
133.19	2	2	2	1	1
161.69	4	3	3	2	3
215.4	3	3	3	2	3
192.64	3	2	2	2	3
176.11	4	3	3	2	3
185	3	3	3	3	2
155.3	2	2	2	2	3
147	3	3	2	2	2
182.98	3	3	3	2	2
238.4	3	3	2	2	3
205.8	4	3	3	2	3
161.48	2	2	2	1	1

DA_7030	DA_7050	DA_7070
5	4	3
4	4	4
4	4	2
3	3	2
3.5	2	2
5	4	3
4	3	3
3.5	3	3
4	3	3
4	4	3
3	3	2
3	2	2
3	4	3
4	4	3
2	2	1
4	3	2
4	4	3
3	3	2
3	2	2
5	4	3
4	3	3
4	4	3
4	4	4
4	3	2
3	3	2
3	2	2
4	4	3
4	3	3
4	3	3
4	4	3
4	4	2
4	4	3
4	4	3
2	2	1
4	3	3
4	4	3
4	3	2
4	4	3
3	3	2
4	3	2
3	3	4
3	2	2
4	3	3
5	4	3
2	2	1
